# Resting Tendon Cross-Sectional Area Underestimates Biceps Brachii Tendon Stress: Importance of Measuring During a Contraction

**DOI:** 10.3389/fphys.2021.654231

**Published:** 2021-09-27

**Authors:** Rowan R. Smart, Brian O'Connor, Jennifer M. Jakobi

**Affiliations:** ^1^Healthy Exercise and Aging Laboratory, School of Health and Exercise Sciences, University of British Columbia Okanagan, Kelowna, BC, Canada; ^2^Department of Psychology, Faculty of Arts and Social Sciences, University of British Columbia Okanagan, Kelowna, BC, Canada

**Keywords:** ultrasound, aging, tendon mechanics, *in vivo*, elbow flexion, sex differences

## Abstract

Force produced by the muscle during contraction is applied to the tendon and distributed through the cross-sectional area (CSA) of the tendon. This ratio of force to the tendon CSA is quantified as the tendon mechanical property of stress. Stress is traditionally calculated using the resting tendon CSA; however, this does not take into account the reductions in the CSA resulting from tendon elongation during the contraction. It is unknown if calculating the tendon stress using instantaneous CSA during a contraction significantly increases the values of *in vivo* distal biceps brachii (BB) tendon stress in humans compared to stress calculated with the resting CSA. Nine young (22 ± 1 years) and nine old (76 ± 4 years) males, and eight young females (21 ± 1 years) performed submaximal isometric elbow flexion tracking tasks at force levels ranging from 2.5 to 80% maximal voluntary contraction (MVC). The distal BB tendon CSA was recorded on ultrasound at rest and during the submaximal tracking tasks (instantaneous). Tendon stress was calculated as the ratio of tendon force during contraction to CSA using the resting and instantaneous measures of CSA, and statistically evaluated with multi-level modeling (MLM) and Johnson–Neyman regions of significance tests to determine the specific force levels above which the differences between calculation methods and groups became statistically significant. The tendon CSA was greatest at rest and decreased as the force level increased (*p* < 0.001), and was largest in young males (23.0 ± 2.90 mm^2^) followed by old males (20.87 ± 2.0 mm^2^) and young females (17.08 ± 1.54 mm^2^) (*p* < 0.001) at rest and across the submaximal force levels. Tendon stress was greater in the instantaneous compared with the resting CSA condition, and young males had the greatest difference in the values of tendon stress between the two conditions (20 ± 4%), followed by old males (19 ± 5%), and young females (17 ± 5%). The specific force at which the difference between the instantaneous and resting CSA stress values became statistically significant was 2.6, 6.6, and 10% MVC for old males, young females, and young males, respectively. The influence of using the instantaneous compared to resting CSA for tendon stress is sex-specific in young adults, and age-specific in the context of males. The instantaneous CSA should be used to provide a more accurate measure of *in vivo* tendon stress in humans.

## Introduction

Producing movement and torque around a joint requires the transfer of force from the muscle to the bone *via* the tendon. As the muscle shortens, forces placed on the tendon are distributed through the cross-sectional area (CSA) of the tendon, and this ratio of force to the CSA is quantified as the tendon's mechanical property of stress (Vergari et al., 2011; Eriksen et al., 2018; Lepley et al., 2018; Ristaniemi et al., 2018; Smart et al., 2018a). Stress is dependent on the amount of force applied to the tendon and the CSA of the tendon, such that greater amounts of applied force and smaller tendon CSA culminate in the higher stress.

Human *in vivo* studies evaluating the tendon stress traditionally rely on the measure of engineering stress which assumes that CSA is constant from rest to the maximal forces, and uses the resting tendon CSA for all the calculations of stress (Stenroth et al., 2012; Eriksen et al., 2018; Lepley et al., 2018). This experimental approach is appealing, as resting CSA measures are relatively easy to acquire (Stenroth et al., 2012; Eriksen et al., 2018). Since the tendon CSA decreases as the applied force increases (Vergari et al., 2011; Obst et al., 2014b; Smart et al., 2018b), utilizing this singular resting CSA measure for all the contraction intensities likely underestimates the tendon stress, and the magnitude of the underestimation is likely greater at higher relative forces when the decrease in tendon CSA is the greatest (Smart et al., 2017, 2018a,b). Therefore, applying resting CSA in the calculation of tendon stress fails to account for the dynamic nature of the tendon and likely misrepresents the true amount of stress experienced by the tendon across various force levels.

In an *ex vivo* equine tendon model, Vergari et al. (2011) demonstrated that when increasing the tendon strain (elongating the tendon by a percentage of its resting length), CSA decreased linearly while the stress increased in a non-linear fashion. Over the tested strain levels, Vergari et al. (2011) found that the tendon stress was 7–14% greater when calculated using the instantaneous compared with resting CSA. This underestimation that occurs when resting CSA is used, has not been investigated *in vivo* for human tendons. The technique of *ex vivo* studies evaluating the tendon stress as the tendon is lengthened to a given amount of strain is not experimentally viable for *in vivo* human tendons. Notwithstanding the potential to use multiple ultrasound probes, which in some tendons is anatomically impossible, this technique would require real-time monitoring of the tendon elongation and CSA during the contraction. Current two-dimensional (2D) ultrasound technology does not allow for real-time simultaneous recordings of *in vivo* elongation and CSA.

To understand the effects of strength and tendon size on the increase in *in vivo* tendon stress values from the resting to instantaneous CSA calculation, we evaluated the sex- and age-group differences at relative force levels. Prior studies of the Achilles tendon showed greater stress in young compared with old (Stenroth et al., 2012) and males compared with females (Stenroth et al., 2012; Lepley et al., 2018) when calculated using the resting tendon CSA, and we showed that distal BB tendon stress is greater in young males as compared with old males when calculated using the instantaneous CSA (Smart et al., 2018a). Young males are stronger than young females and old males (Pereira et al., 2015; Smart et al., 2018a), and have greater distal biceps brachii (BB) tendon elongation and CSA than the old males (Smart et al., 2018a) and presumably young females. The higher strength in young males would generate greater pull on the tendon and augment elongation and CSA reductions at relative force levels, increasing the difference in tendon stress values between the resting and instantaneous CSA calculations to a greater extent than the old males and young females.

The purpose of this study was to compare calculations of tendon stress values for the distal BB tendon using the resting and instantaneous measures of tendon CSA across different force levels. We evaluated sex- and age-related differences in the two measures of tendon stress with the multi-level statistical modeling procedures. The use of modeling is beneficial for answering the unique research questions across multiple research fields, such as neuromuscular physiology (Taylor and Enoka, 2004; Tibold and Fuglevand, 2015; Potvin and Fuglevand, 2017). We hypothesized that the tendon CSA would decrease during a contraction due to tendon elongation, leading to higher tendon stress calculated using the instantaneous CSA compared with the resting CSA and that the difference would be greater at higher forces. Second, we hypothesized that due to a combination of greater strength and the decrease in tendon CSA, the difference in tendon stress values from the resting to instantaneous CSA conditions would be highest in young males, followed by old males and young females, and that the difference between stress values would become statistically significant at lower forces in young males.

## Materials and Methods

### Participants

Tendon stress was determined for nine young males and nine old males using a previously published data set (Smart et al., 2018a), and for eight young females using unpublished data (Table 1). The experimental set-ups and procedures were identical for both datasets apart from the submaximal force levels as described in the protocol section below. All participants were recreationally active, and the old males were non-frail, community dwelling, and functionally independent. Most spoke of engagement with outdoor activities, such as gardening and golfing. All the participants self-identified as right-hand dominant. The exclusion criteria for this study were orthopedic surgery or injury to the right shoulder or arm in the prior 6 months, participation in high levels of upper body strength training, or conditions that influence the muscle and tendon beyond typical age-related changes. Written informed consent was obtained from all participants prior to their participation and ethics approval was granted from the University of British Columbia Okanagan's Behavioral Research Ethics Board.

**Table 1:** Subject characteristics.

	**Young-males**	**Old-males**	**Young-females**	**Group effect**
Age (yrs)	22 ± 1^#^	76 ± 4^*^	21 ± 1^#^	*F*_(2,25)_ = 849.66, η^2^ = 0.99, *p* < 0.01
Height (cm)	180.0 ± 7.0	173.9 ± 6.9	167.0 ± 7.6^*^	*F*_(2,25)_ = 7.06, η^2^ = 0.38, *p* < 0.01
Body mass (kg)	79.4 ± 8.3	78.0 ± 10.9	61.0 ± 5.5^*^^#^	*F*_(2,25)_ = 11.68, η^2^ = 0.50, *p* < 0.01
MVC (N)	250.7 ± 31.0^#^	200.1 ± 28.5^*^	149.8 ± 27.0^*^^#^	*F*_(2,25)_ = 25.7, η^2^ = 0.69, *p* < 0.01
Moment arm (mm)	56.1 ± 4.7	55.9 ± 4.1	53.6 ± 4.6	*H*_(2)_ = 3.98, *p* = 0.14
Lever arm (mm)	354.3 ± 11.6	348.6 ± 16.1	322.3 ± 18.9^*^^#^	*F*_(2,25)_ = 9.92, η^2^ = 0.46; *p* < 0.01
Stiffness (N/mm)	170 ± 132.9	113.0 ± 55.1	55.8 ± 43.3^*^	*H*_(2)_ = 6.857, *p* = 0.032

*MVC, maximal voluntary contraction; N, Newtons*.

* *Differs from young males*;

# *differs from old males. Values are mean ± SD*.

### Experimental Set-Up

**Figure 1:**
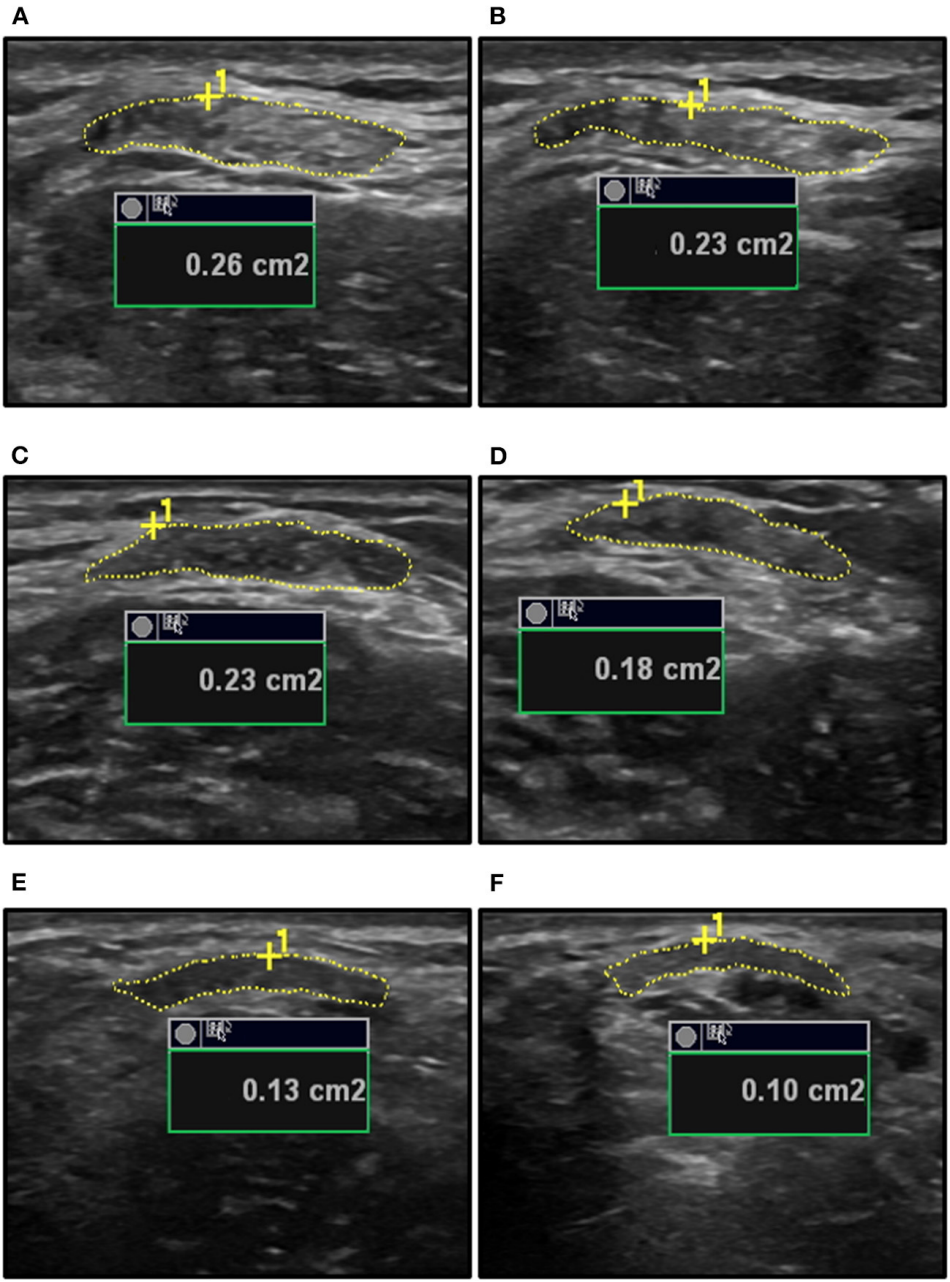
The ultrasound measurements of biceps brachii (BB) tendon cross-sectional area (CSA) for a young male at rest **(A)** and during a 20% MVC contraction **(B)**, an old male at rest **(C)** and during a 20% MVC contraction **(D)**, and a young female at rest **(E)** and during a 25% MVC contraction **(F)**. The inherent measurement platform of the ultrasound provides the area in square centimeters (cm^2^) and this was converted to square millimeter (mm^2^) prior to the calculations of tendon stress using the resting and the instantaneous tendon CSA measures. MVC, maximal voluntary contraction.

**Figure 2:**
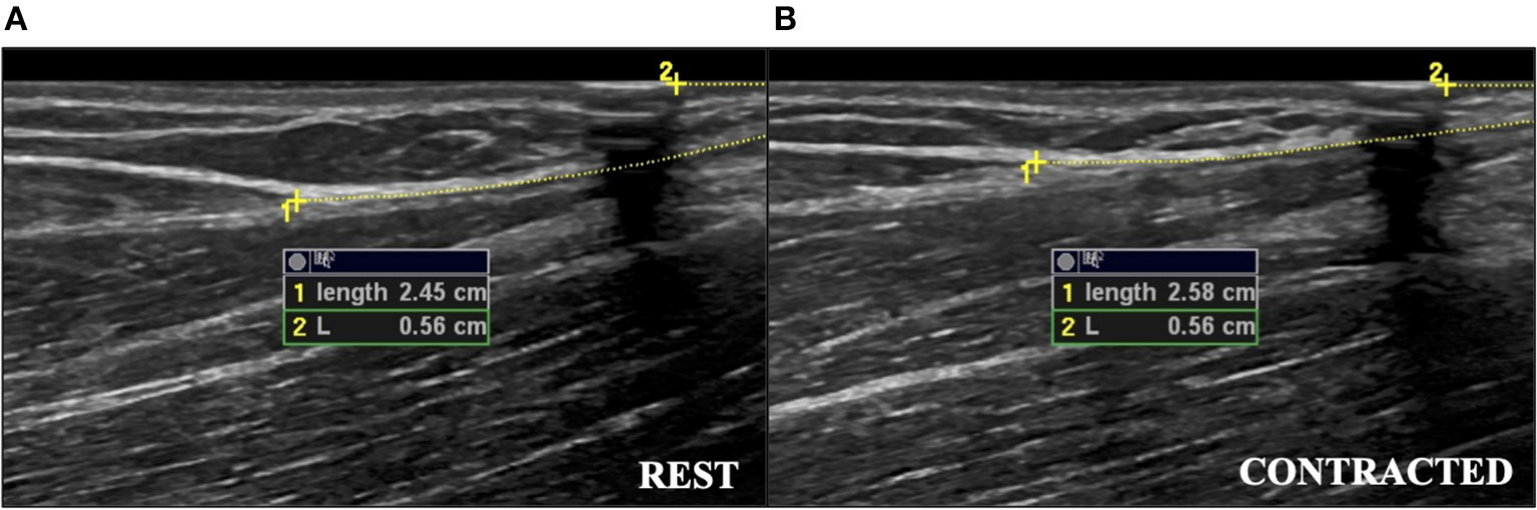
The ultrasound measurements of tendon elongation in a young female during a 25% MVC contraction. **(A)** Measurement at rest of distance from muscle-tendon junction to edge of ultrasound field of view (Length 1), and measurement of distance from hyperechoic marker to the edge of ultrasound field of view (L2). **(B)** Measurement during the contracted state. The difference between the measurements during the contracted and resting states was used as the measure of tendon elongation. Images have been cropped for reproduction, but analysis captured the full length of the tendon. MVC, maximal voluntary contraction.

Participants were seated in a custom-built isometric dynamometer chair (Brown et al., 2010; Harwood et al., 2010; Smart et al., 2017, 2018a,b) with their right arm positioned at 110° of elbow flexion (full extension being 180°), the shoulder forward flexed 15°, and the right hand grasping the manipulandum with the wrist in a neutral position halfway between full supination and pronation. The force transducer (MLP-150, Transducer Techniques, Temecula, CA, USA) was located directly below the hand. Force was sampled at 2,381 Hz, converted from analog-to-digital format using a power 1401 plus [Cambridge Electronic Designs (CED), Cambridge, England], and stored for offline analysis using Spike 2 v7.12 (CED, Cambridge, England). The real-time visual feedback of the force signal was displayed on a 52 cm monitor positioned 1-m in front of participants. A B-mode ultrasound probe (ML6-15, 4–15 MHz, GE LOGIQ E9; General Electric, Fairfield, CT, USA) was placed in a custom-made probe holder and secured over the distal BB tendon. The probe was positioned in transverse orientation at the mid-point of the BB tendon to visualize the tendon in cross-section (Figure 1). For tendon elongation, the probe was secured in a longitudinal orientation to visualize the muscle-tendon junction (MTJ) of the long head of the BB (Figure 2). Visual representation of the experimental set-up has been previously published (Smart et al., 2017).

### Anatomical Measures

Anatomical measures were performed as previously described (Smart et al., 2017, 2018a,b). Briefly, lever arm length was measured on the skin surface as the distance from the head of the radius to the force transducer. The moment arm was obtained by locating the distal MTJ of the BB and the insertion of the distal BB tendon onto the radius using ultrasound and indicating these points on the skin. A linear edge was placed between these points and the moment arm was measured as the perpendicular distance from this linear edge to the lateral epicondyle of the humerus.

### Protocol

Participants performed three isometric elbow flexion maximal voluntary contractions (MVCs). In the experiment involving young and old males, the submaximal forces of 2.5, 5, 10, 20, 40, 60, and 80% MVC were calculated, while force levels of 5, 10, 25, 50, and 75% MVC were used in the study of young females. The submaximal contractions consisted of a 3-s ramp to the target force level, a 5–10 s plateau, and a 3-s ramp down to the baseline. Following each contraction, 2-min rest was provided to ensure that there was no fatigue. The feedback of the force signal during the submaximal contractions was scaled according to the MVC of the participant to provide similar visual feedback across participants and force levels (Harwood et al., 2014). Ultrasound videos of tendon CSA (Figure 1) and elongation were recorded during the contractions at a frame rate of 31 Hz. Each submaximal contraction level was performed four times to obtain the two recordings of tendon CSA and two of elongation. The force levels were randomized within the CSA and elongation blocks, and the average of the two contractions are reported for elongation, tendon force, CSA, and stress. Following the submaximal contractions, participants performed a final MVC that was within 5% of their initial MVC to ensure fatigue did not occur as a result of the protocol.

### Data Analysis

Tendon CSA and elongation were captured using ultrasonography through the video recordings of the tendon in a cross-sectional or longitudinal view, respectively. Tendon CSA was measured at rest and at the mid-point of the steady-state plateau of the submaximal contractions by tracing the outer border of the tendon using the inherent measurement tool platform of the ultrasound (GE LOGIQ E9). For tendon elongation, it was measured as the difference in length from the distal BB MTJ to the edge of the ultrasound field of view in the resting state and the mid-point of the plateau in the contracted state. The distance to a hyperechoic marker was also measured to ensure that the probe did not move during the contractions (Smart et al., 2017, 2018a,b). The high repeatability of ultrasound measurements for the distal BB tendon has been previously published by our lab group (Smart et al., 2017). The force was analyzed as the mean force produced during the middle 3–5 s of the plateau phase of the contractions. The tendon stiffness was calculated as detailed in the prior studies on the distal BB tendon (Smart et al., 2018a,b) as the slope of the tendon force/elongation relationship between low (~20%) and high (~80%) submaximal forces. Tendon stress was calculated at each force level using the resting and instantaneous CSA. Following determination of the muscle moment (Equation 1), tendon force was computed using moment arm length (Equation 2). This was subsequently used in the calculation of tendon stress (Equation 3) for the two conditions of resting and instantaneous tendon CSA. The calculation of tendon stress has been described for various tendons using resting (Stenroth et al., 2012; Eriksen et al., 2018; Lepley et al., 2018) and instantaneous CSA (Smart et al., 2017, 2018a,b).


(1) $$\begin{array}{r} \text{Force}\left(N\right)*Lever\;arm\;length\left(mm\right)=Muscle\;moment\left(N*mm\right) \end{array}$$

(2) $$\begin{array}{r} \frac{Muscle\;moment\left(N*mm\right)}{Moment\;arm\;length\left(mm\right)}=Tendon\;force\left(N\right) \end{array}$$

(3) $$\begin{array}{r} \frac{Tendon\;force\left(N\right)}{Tendon\;CSA\left(mm^{2}\right)}=Tendon\;stress\left(MPa\right) \end{array}$$

### Statistical Analysis

#### Tests of Normality and Mixed-Model ANOVAs

The tests of normality were conducted for all variables. Shapiro–Wilk's test indicated that the moment arm and tendon stiffness were not normally distributed for old males (*p* = 0.020, *p* = 0.024) and young females (*p* = 0.003, *p* = 0.035). In young males, the moment arm was not normally distributed (*p* = 0.032) and the tendon stiffness approached significance (*p* = 0.061) in not being normally distributed. For non-normally distributed variables, the Kruskal–Wallis test was used to compare the groups. For significant effects, *post-hoc* Mann–Whitney *U-*tests were applied to determine the differences between the groups. MVC and lever arm were compared between the groups using one-way ANOVAs with Tukey's *post-hoc* tests. In order to compare among all the three groups and across all force levels, tendon CSA was evaluated using a 3 (group: young males, old males, young females) × 11 (resting, 2.5, 5, 10, 20, 25, 40, 50, 60, 75, and 80% MVC) mixed-model ANOVA, while tendon force and elongation were evaluated with a 3 (group: young males, old males, young females) × 10 (force level: 2.5, 5, 10, 20, 25, 40, 50, 60, 75, and 80% MVC) ANOVA. When significant interactions occurred, one-way ANOVAs with Tukey's *post-hoc* tests were used to compare among the groups at each force level. The statistical analyses for the ANOVAs were performed using SPSS version 25 (IBM, Amrok, NY, USA). The effect sizes are reported as eta squared (η^2^) for one-way ANOVAs, and partial eta squared ($\eta_{\text{p}}^{2}$) for the two-way ANOVAs. Data are reported as means ± SD, and the alpha level was set at 0.05.

#### Multi-Level Modeling and Johnson–Neyman Analysis

The data were also evaluated using growth curve analyses *via* multi-level modeling (MLM), which allows comparisons among the groups when the levels of repeated measurements are not the same for all persons. The analyses also provided a broader picture of the differences in tendon stress across conditions (the resting or instantaneous CSA), sex-age, and force levels.

The MLM growth curve analyses of tendon stress were conducted using the nlm package in R (Pinhero and Bates, 2000; Snijders and Bosker, 2012; Verbeke, 2013; Hox et al., 2018; Humphrey and LeBreton, 2019). In this procedure, a curve was fit for every participant for the relationship between force level (on the *x*-axis) and tendon stress (on the *y*-axis). The tests were then conducted to determine whether there was significant variation in the intercepts and slopes of the curves. When there was significant variation in either of these parameters, the predictor variables were introduced in an effort to account for the variation. The first predictor variable (sex-age) was examined as a categorical variable representing the three groups: young males, old males, and young females. The second predictor variable was instantaneous-resting, representing the two tendon CSA states. After testing for interactions between the predictor variables in the MLM, the Johnson–Neyman regions of significance procedure (Johnson and Fay, 1950; Bauer et al., 2005; Lazar and Zerbe, 2011; Rast et al., 2014; Hayes, 2018) was conducted to identify the precise force levels at which there were statistically significant differences in tendon stress between the two resting-instantaneous conditions and among the three sex-age groups within the resting and instantaneous conditions, with the probability of significance placed at 0.05.

The use of MLM and the Johnson–Neyman regions of significance procedure are novel statistical procedures in the study of tendon mechanics. They provide a more precise determination, compared with the traditional methods, of the point at which group and condition differences begin to occur, based on the simultaneous analyses of all available data.

## Results

### Subject Characteristics

Young males were the strongest followed by old males and young females (*p* < 0.01). There was no difference in the tendon moment arm among the groups (*p* = 0.14). The lever arm did not differ between young and old males but was shorter in young females compared with both the young and old males (*p* < 0.01). Tendon stiffness did not differ between young and old males (*p* = 0.49) and between old males and young females (0.064) but was significantly greater in young males compared with young females (*p* = 0.012) (Table 1).

### Multi-level Modeling for Resting and Instantaneous Tendon CSA

The MLM method of testing for the significant differences between the lines, and for group differences in the slopes of the lines, involves examining the fit levels for a sequence of models. The likelihood ratio (LR) test established that permitting the intercepts to vary across participants did not improve the model fit (LR = 0.00000048, *p* = 0.9994), and therefore, the intercepts were fixed (not permitted to vary) for all the subsequent analyses. In contrast, permitting the slopes to vary across participants did increase the model fit relative to a fixed slope model, LR = 1091.35, *p* < 0.0001. This means that there was statistically significant variation in the slopes, but not in the intercepts. A model with the interaction between the sex-age predictor and force level for the slopes fit the data better than a model without the interactions (LR = 76.7, *p* < 0.0001). Similarly, for the instantaneous-resting predictor, a model that included the interaction between instantaneous-resting and force level fit the data more appropriately (LR = 56.83, *p* < 0.0001) than a model that did not include the interaction, and thus both the expected two-way interactions were significant. Finally, a test for the possible three-way interaction (young males, old males, and young females × instantaneous-resting × force level) indicated an improvement in fit over a model that contained all the possible two-way interactions, LR = 479.65, *p* < 0.0001, but none of the individual three-way terms in the model were significant. This was likely due to the small sample size for the three-way interactions. The subsequent analyses, therefore, focused on the two-way interactions.

### Johnson–Neyman Regions of Significance

**Figure 3:**
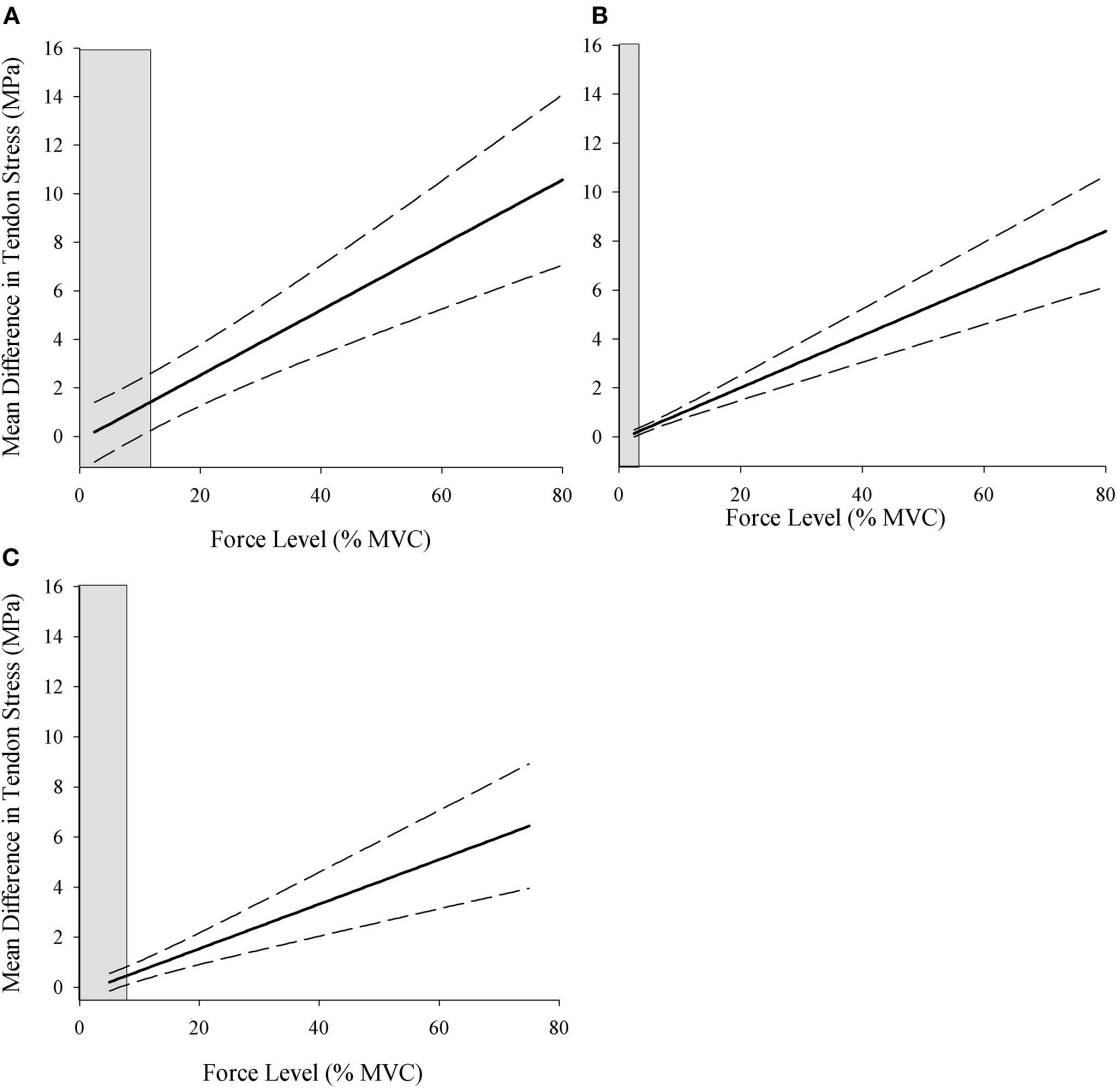
Johnson–Neyman regions of significance for the difference in stress calculated using the resting and instantaneous CSA for young males **(A)**, old males **(B)**, and young females **(C)**. Values to the right of the shaded areas indicate the statistically significant differences between stress values calculated using the resting and instantaneous tendon CSA (*p* < 0.05). MVC, maximal voluntary contraction; MPa, megapascals.

The Johnson–Neyman regions of significance analysis was conducted to determine the force-level above which the difference values for tendon stress became statistically significant for comparisons between (1) the instantaneous and resting CSA conditions for each of the three sex-age groups, and (2) each of the three sex-age groups within the resting and instantaneous conditions separately. The differences between stress calculated with the resting and instantaneous CSA are plotted across the force levels for young males, old males, and young females (Figure 3), and the mean difference was greatest in young males (0.64–11.19 MPa), followed by old males (0.15–9.3 MPa), and young females (0.21–7.37 MPa).

The difference value at the relative force level where the shaded gray area ends is the point at which the stress values become significantly different between the resting and instantaneous CSA calculations for each sex-age group. The shaded gray area on the left side of the plots represents the region of non-significance, and the non-shaded area is the region of significant differences between the calculations. The region of significance began at lower force levels in old males (>2.56% MVC) followed by young females (>6.63% MVC) and young males (>9.98% MVC) (Table 2, Figure 3), indicating that the point at which tendon stress is significantly greater using the instantaneous CSA is both age- and sex-specific.

**Table 2:** Johnson–Neyman regions of significance.

	**Comparison**	**Force level (% MVC) at which differences in stress become significantly different**
Young-males	Resting vs. instantaneous	9.98
Old-males	Resting vs. instantaneous	2.56
Young-females	Resting vs. instantaneous	6.63
Resting	Young-males vs. old-males	9.94
Resting	Young-males vs. young-females	3.47
Resting	Old-males vs. young-females	1.91
Instantaneous	Young-males vs. old-males	5.95
Instantaneous	Young-males vs. young-females	2.64
Instantaneous	Old-males vs. young-females	15.07

The Johnson–Neyman analysis also evaluated the group differences in tendon stress within the resting and instantaneous CSA conditions separately in order to better understand the influence of tendon condition on the population differences in tendon stress. This comparison evaluated the differences between the groups within the resting and instantaneous CSA conditions separately. In the resting CSA condition, young males had significantly greater tendon stress than old males at 9.94% MVC and above. However, this threshold decreased to 5.95% MVC in the instantaneous CSA condition, highlighting that the age-related difference in stress between the young and old males became statistically significant at a lower relative force level when stress was calculated from the instantaneous CSA measure. Similarly, young males had greater tendon stress than young females above 3.47% MVC in the resting CSA condition, and this decreased to 2.64% MVC in the instantaneous condition, and thus the sex-related difference in tendon stress also occurred at a lower force when the instantaneous CSA is used in the calculation. The decrease in significance threshold for the age- and sex comparisons demonstrates that the higher stress values in the instantaneous condition led to the significant group differences in tendon stress at lower relative force levels. However, the comparison between old males and young females did not follow this expected trend. The threshold actually increased from 1.91 to 15.07% MVC between the resting and instantaneous calculations, and this is in part due to data variability as well as the physiological oddity of comparing young females to old males (Table 2).

### Supplemental ANOVA Results

**Figure 4:**
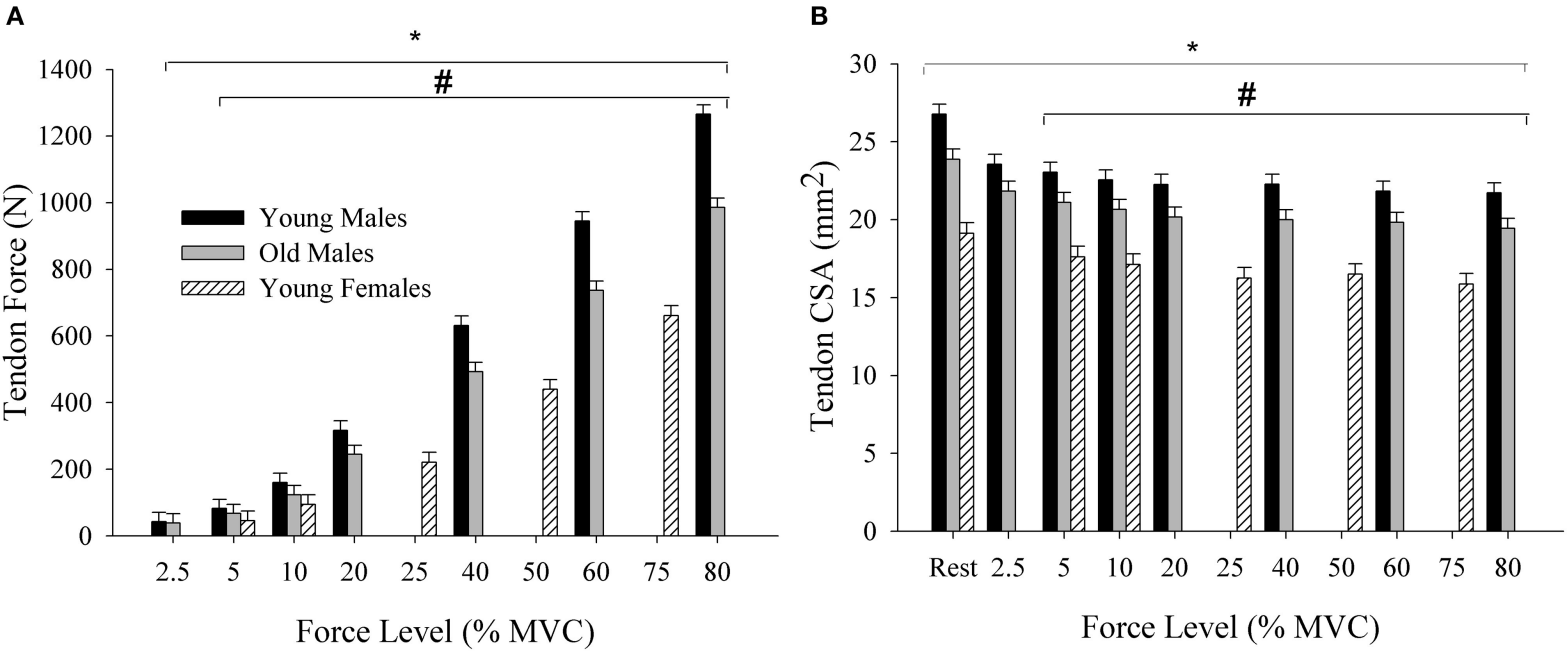
**(A)** Tendon force across the submaximal force levels for young and old males and young females. Tendon force increased with the contraction intensity and was greater in young males compared with the old males and young females. **(B)** Tendon CSA at rest and across the submaximal force levels. CSA, cross-sectional area; MVC, Maximal voluntary contraction; N, Newtons. *differs across force levels; ^#^differs between all the three groups at 5% and 10% MVC, and between young and old males at 20, 40, 60, and 80% MVC (*p* < 0.05).

There was a force level by group interaction for tendon force [*F*_(7,147)_ = 6.34; $\eta_{\text{p}}^{2}$ = 0.22; *p* < 0.001]. Tendon force increased from 2.5% MVC (40.83 ± 6.65 N) to 80% MVC (1126.22 ± 221.40 N) [force main effect: *F*_(9,147)_ = 340.63; $\eta_{\text{p}}^{2}$ = 0.95; *p* < 0.001], and the interaction occurred from the tendon force being the greatest in young males (492 ± 80.75 N), followed by old males (384.3 ± 51.4 N) and young females (292.66 ± 42.86 N) (*p* < 0.001) at 5 and 10% MVC, and greater in young males than the old males from 20 to 80% MVC (Figure 4A). Tendon CSA had main effects of force [*F*_(10,169)_ = 9.01; $\eta_{\text{p}}^{2}$ = 0.35; *p* < 0.001] and group [*F*_(2,169)_ = 78.85; $\eta_{\text{p}}^{2}$ = 0.48; *p* < 0.001]. CSA values during the active muscle contraction decreased across the submaximal force levels, and was greatest in young males (23.0 ± 2.90 mm^2^) followed by old males (20.87 ± 2.0 mm^2^) and young females (17.08 ± 1.54 mm^2^) (*p* < 0.001) (Figure 4B). There was a force by group interaction for elongation [*F*_(7,152)_ = 2.01; $\eta_{\text{p}}^{2}$ = 0.099; *p* = 0.049]. The tendon elongated 14.1 mm (1.37 ± 0.82 to 15.47 ± 4.38 mm) in young males, 8.81 mm (2.45 ± 1.27 to 11.26 ± 3.50 mm) in old males, and 6.21 mm (2.50 ± 2.58 to 8.79 ± 7.51 mm) in young females [*F*_(9,152)_ = 23.77; $\eta_{\text{p}}^{2}$ = 0.62; *p* < 0.001]. The elongation did not differ among the three groups at 5% MVC (2.78 ± 1.94 mm, *p* = 0.11) and 10% MVC (3.98 ± 2.42 mm, *p* = 0.23), but was greater in young males than the old males at 60% MVC (13.88 ± 4.15, 9.78 ± 2.27 mm, *p* = 0.028) and 80% MVC (15.47 ± 4.38, 11.26 ± 3.50 mm, *p* = 0.047).

## Discussion

The present study showed significant variation in the rate of increase in distal BB tendon stress values among young males, old males, and young females as well as between the resting and instantaneous CSA conditions, and that the tendon stress values were significantly greater in the instantaneous compared with the resting CSA condition. The use of MLM and Johnson–Neyman regions of significance tests allowed for a more precise and comprehensive analysis of the tendon stress data to establish the specific force levels of statistical differences, regardless of the submaximal force levels performed. The Johnson–Neyman regions of significance tests showed that (1) the difference between the resting and instantaneous CSA stress values became greater as the submaximal force levels increased, (2) this difference was greatest in young males, followed by old males and young females, and (3) sex- and age-group differences in tendon stress occurred at lower relative forces when instantaneous CSA was used in the calculations. Overall, calculating the distal BB tendon stress with resting CSA significantly underestimated *in vivo* tendon stress compared to the calculations using CSA measured during the muscle contraction, and the extent of underestimation was both age- and sex-specific.

### Tendon Stress Across Submaximal Forces

**Figure 5:**
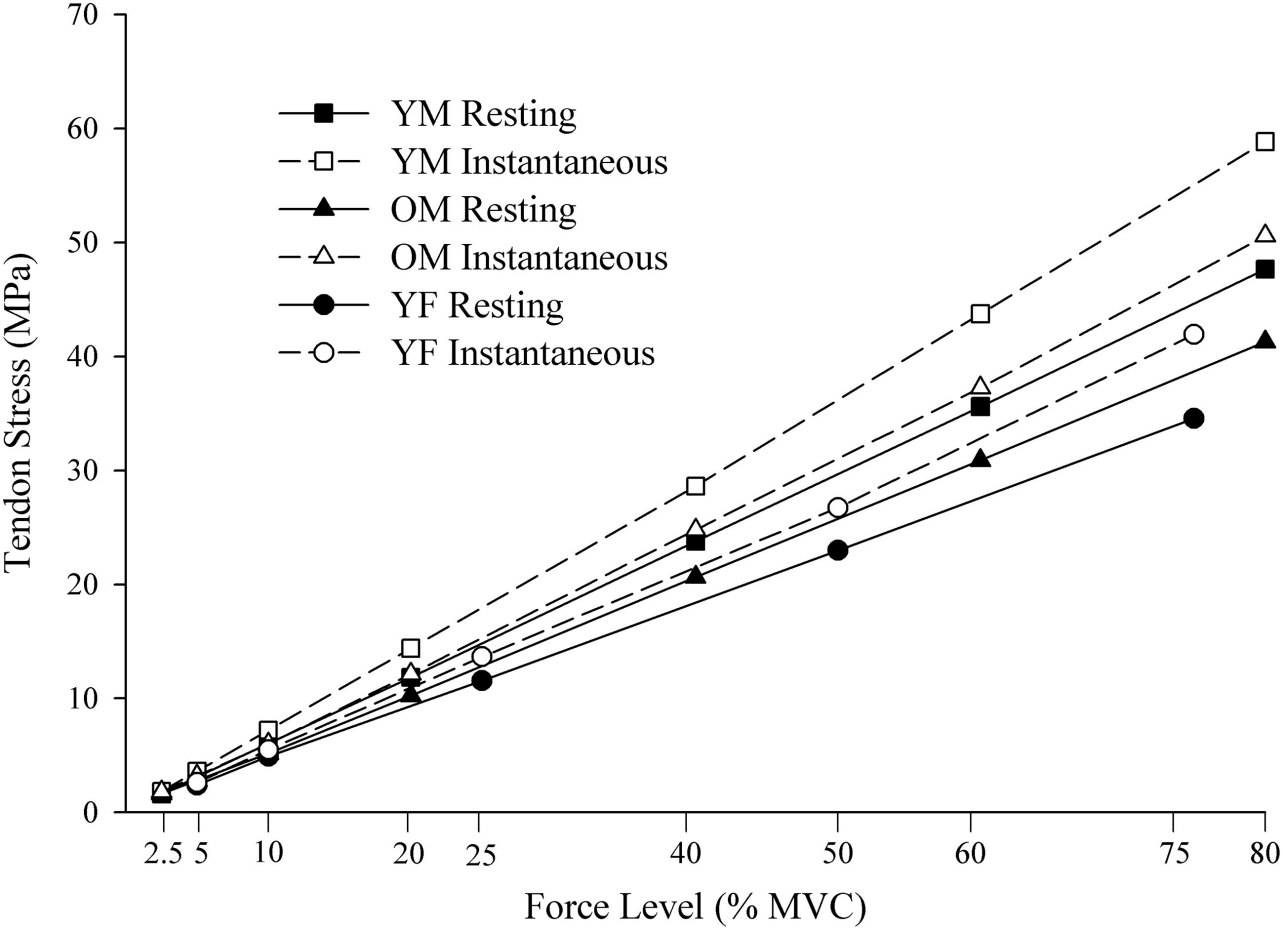
Stress values calculated using the resting and instantaneous tendon CSA. CSA, cross-sectional area YM, young males; OM, old males; YF, young females. MPa, megapascals; MVC, maximal voluntary contraction.

The MLM growth curve analyses were beneficial in determining the rate of increase in distal BB tendon stress values across the relative force levels was greater using the instantaneous compared with the resting CSA, and that young males had the fastest rate of increase followed by old males and young females (Figure 5). The condition and group differences in tendon stress were evident through an improvement in model fit of the slopes when interaction terms were included for the instantaneous-resting predictors and sex-age predictors. The increase in slope of tendon stress from the resting to instantaneous condition resulted from the progressive decrease in CSA due to the constant volume of tendon being lengthened as the submaximal force increased. This contributed to the ratio of tendon force to CSA becoming greater at a faster rate in the instantaneous compared with the resting condition. The decrease in CSA resulting from tendon lengthening has been previously reported for the distal BB tendon (Smart et al., 2018a,b), Achilles tendon (Obst et al., 2014b), as well as *ex vivo* equine tendon (Vergari et al., 2011). The age- and sex-related differences in the rate at which tendon stress increased across the force levels resulted from young males being the strongest, followed by old males and young females. The greater strength in young males led them to apply more absolute force to the tendon at the relative force levels, culminating in greater tendon force in young males, followed by old males and young females. When combined with the reduction in CSA, the higher tendon force in young males contributes to a faster rate of increase in tendon stress values (steeper slope), followed by old males and young females.

### Resting and Instantaneous Tendon CSA

In support of the first hypothesis, the Johnson–Neyman analysis demonstrated that tendon stress differed between the resting and instantaneous CSA calculations within each group. Old males had the lowest relative force level at which differences between the resting and instantaneous CSA stress calculations became statistically significant, followed by young females and young males (Table 2, Figure 3). This does not align with the second hypothesis as we expected that young males would show statistical differences at lower forces because they were the strongest and would have the largest ratio of tendon force to CSA. However, old males had a greater decrease in tendon CSA (5%) at the lower force levels (2.5–10%) compared with young females (3%) and young males (4%) (see Figure 4), and this likely amplified the ratio of tendon force to the instantaneous CSA leading to significantly greater stress at the lower force levels in the old males. The order effect of the groups in the determination of tendon stress between calculation methods emphasizes the importance of considering the study sample under investigation. The comparison between age or sex groups, for example, provides an opportunity to consider the contribution of population differences in the mechanical (Stenroth et al., 2012; Lepley et al., 2018; Smart et al., 2018a) and material (Thorpe et al., 2017) properties of the tendon to CSA. A prior study of the medial gastrocnemius of young and old females showed that fascicle slack and tendon stiffness were greater in young females compared with old (Csapo et al., 2014). If young males of the present study exhibited greater fascicle slack for the BB, fascicles would need to shorten to a greater extent prior to the tendon lengthening. The greater fascicle slack in young males, combined with their stiffer tendon, could explain why their tendon CSA did not decrease as much at the lower force levels and led to significantly greater tendon stress in the instantaneous condition occurring at a higher relative force level; however, sex- and age-related differences in the BB muscle slack and tendon stiffness require further study.

### Sex- and Age-Related Differences in Stress Underestimation

In support of the second hypothesis, young males had the greatest increase in stress values between the resting and instantaneous CSA calculations, followed by old males and young females (Figure 5). In the resting CSA condition, the sex and age-related differences in tendon stress resulted from young males being the strongest, followed by old males and young females, and this aligns with prior reports on the age-related and sex-specific differences in elbow flexion strength (Brown et al., 2010; Pereira et al., 2015; Smart et al., 2018a). The greater strength in young males resulted in more absolute force being placed on the tendon at the submaximal relative force levels, and this generated a higher ratio of tendon force to CSA. The higher applied forces in young males would also lead to greater reductions in tendon CSA relative to rest, thereby, further amplifying the difference in the resting and instantaneous CSA stress values (the ratio of tendon force to CSA).

When comparing tendon stress calculated with the resting CSA across the three groups and comparing tendon stress calculated with the instantaneous CSA across the three groups, tendon stress in young males became statistically greater than the old males and young females at a lower relative force level in the instantaneous compared with the resting CSA condition (Table 2). This was likely due to the decrease in CSA amplifying the rate of increase in tendon stress values, creating larger group differences at the lower relative force levels. Overall, the Johnson–Neyman analysis demonstrated that the difference between resting and instantaneous CSA stress values is age and sex-specific. The underestimation of tendon stress arising from the use of resting CSA needs to be contextualized within the population of study and may lead to age- and sex-related differences in the tendon stress being overlooked or erroneously diminished.

The underestimation of tendon stress generated by the use of resting CSA in the calculation has also been shown in an *ex vivo* equine tendon model by Vergari et al. (2011) where stress at tendon failure was 10.9% greater using the instantaneous compared with the resting CSA. Our findings in humans show ~20% higher tendon stress during the submaximal contractions of 75–80% MVC, when instantaneous CSA was used, suggesting that failure stress for human tendons is likely higher than previously reported (Lewis and Shaw, 1997; Wren et al., 2001). Although the differences in stress we observed between the instantaneous and resting calculations (0.15–11.19 MPa) were small relative to the total stress experienced by the tendon (41.28–58.84 MPa), it may be consequential to production of steady elbow flexion force control (Smart et al., 2018a), tendon injury, and rehabilitation. Not accounting for the decrease in tendon CSA that occurs during the muscle contraction could lead to excessive stress on the tendon and increase the risk of tendon injury, and the underestimations shown in the present study suggests that this risk of injury may be sex- and age-specific.

### Choices of Statistical Approaches

Multi-level modeling growth curve analysis is a modern, widely recommended analytical method for repeated measures analysis when the measurement levels repeated factors are not exactly the same across persons. Between the two datasets used in the current study, the tendon stress data were obtained at 10 different force levels, however, data were not available for all of the sex-age groups at each of the 10 force levels (young and old males: 2.5, 5, 10, 20, 40, 60, and 80% MVC; young females: 5, 10, 25, 50, and 75% MVC). Using the individual data, growth curves were computed for each participant and further analyses focused on the slopes and intercepts of the computed curves. As the curves were computed for all the three groups using MLM, evaluating the characteristics of the curves among the groups and conditions allowed for comparisons not bound by the individual force levels. MLM also has the benefit of not requiring homoscedasticity within the data, and the heteroscedasticity often present within human physiology data can be incorporated into the MLM analyses *via* weights that are based on the differing variances for more accurate estimates (see Pinhero and Bates, 2000). Models generated in the present study show condition (resting-instantaneous) and sex-age differences for the increase in tendon stress values across the relative forces and highlight the importance of using instantaneous CSA in the determination of stress.

The Johnson–Neyman analysis was used to determine (1) the force levels in which stress values were significantly greater when instantaneous CSA was used compared with the resting CSA within each group, and (2) the force level where stress values became significantly different between groups within the resting and instantaneous CSA conditions. As evidenced by the values in Table 2 and the visual representations in Figure 3, the thresholds for significant differences were not aligned with the submaximal force levels executed and demonstrate that the traditional ANOVA analyses may miss the precise point of the continuous variable at which significant differences start to occur.

## Limitations

From the resting to instantaneous CSA conditions, the higher stress values using instantaneous CSA were expected to lower the force level at which the greater tendon stress in old males became statistically significant from young females, following similar trends as the comparisons to young males. This was not the case as the force level for significant differences between old males and young females increased from the resting to instantaneous CSA conditions, and may have been a result of low sample size increasing variability within the data, as well as this comparison not being physiologically relevant. Future studies examining the influence of resting or instantaneous CSA on the measures of tendon stress should include a sample of old females to allow for a more appropriate sex-related comparison to the old males. To fully appreciate the application of instantaneous compared with the resting tendon CSA, ultrasonography measures should be made along the entirety of the distal BB tendon. Tendon CSA was obtained from the approximate mid-length of the tendon, and may not account for the potential changes in CSA along the entire length of the tendon as seen for the Achilles (Obst et al., 2014a,b) and patellar tendons (Mersmann et al., 2020). Additionally, the measurements of whole BB muscle length along with aponeurosis length would aid in understanding fascicle to aponeurosis ratios of the BB, and future studies should explore this possibility. We performed measures of tendon CSA at relative force levels in contrast to the known strain levels conducted by Vergari et al. (2011). Real-time two-dimensional ultrasound limits the recording to either longitudinal or cross-sectional views making the simultaneous acquisition of tendon elongation and CSA to estimate the stress at a specific strain not feasible. Simultaneous recordings of elongation and CSA could be undertaken in long tendons, e.g., Achilles tendon with two ultrasound probes; however, the anatomy of the elbow limits this possibility.

### Conclusion

The MLM demonstrated that tendon stress values increased at a faster rate across the force levels when calculated using the instantaneous compared with the resting CSA. The difference between resting and instantaneous calculations was greatest in young males followed by old males and young females. Further, statistical modeling through the use of the Johnson–Neyman regions of significance test revealed that the force level at which values became significantly greater in the instantaneous compared with the resting condition depended both on sex and age. These findings suggest that the tendon stress underestimation arising from the use of resting CSA is age- and sex-specific. Future *in vivo* human studies should consider the use of instantaneous tendon CSA for evaluating tendon stress to obtain a true representation of the ability of tendon to distribute force from the muscle to the bone in the production and control of human movement.

## Data Availability Statement

The raw data supporting the conclusions of this article will be made available by the authors, without undue reservation.

## Ethics Statement

The studies involving human participants were reviewed and approved by University of British Columbia Okanagan's Behavioral Research Ethics Board. The patients/participants provided their written informed consent to participate in this study.

## Author Contributions

RS designed study, collected and analyzed data, performed statistical analysis, and prepared manuscript. BO'C performed statistical analysis and prepared manuscript. JJ designed study and prepared manuscript. All authors contributed to the article and approved the submitted version.

## Funding

RS was supported by a Natural Sciences and Engineering Research Council doctoral award and JJ was supported by a Natural Sciences and Engineering Research Council grant (Grant No: 312038).

## Conflict of Interest

The authors declare that the research was conducted in the absence of any commercial or financial relationships that could be construed as a potential conflict of interest.

## Publisher's Note

All claims expressed in this article are solely those of the authors and do not necessarily represent those of their affiliated organizations, or those of the publisher, the editors and the reviewers. Any product that may be evaluated in this article, or claim that may be made by its manufacturer, is not guaranteed or endorsed by the publisher.
